# Digital Technologies Applied to Control the One-Step Process of Cannabis Olive Oil Preparations

**DOI:** 10.3390/pharmaceutics15030870

**Published:** 2023-03-08

**Authors:** Paolo Bongiorno, Antonio Lopalco, Antonella Casiraghi, Antonio Spennacchio, Alessandro Pitruzzella, Angela Assunta Lopedota, Paola Minghetti, Nunzio Denora

**Affiliations:** 1 Farmacia Dott. Paolo Bongiorno, Via Vittorio Veneto 17/19, 92026 Favara, AG, Italy; 2 Department of Pharmacy—Pharmaceutical Sciences, The University of Bari Aldo Moro, Via Orabona 4, 7012 Bari, BA, Italy; 3 Department of Pharmaceutical Sciences, The University of Milan Via G. Colombo 71, 20133 Milan, MI, Italy; 4 Biomedicine, Neuroscience and Advanced Diagnostics, (BIND) University of Palermo, Piazza Marina, 61, 90133 Palermo, PA, Italy

**Keywords:** *Cannabis sativa* L., olive oil formulation, extraction, decarboxylation, cannabinoids, terpenes

## Abstract

The reproducibility of an extemporaneous preparation is an essential condition for guaranteeing the quality, efficacy, and safety of the medicinal product. This study aimed to develop a controlled one-step process for cannabis olive oil preparations by applying digital technologies. For this purpose, the chemical profile of cannabinoid contents in oil extracts of Bedrocan, FM2, and Pedanios varieties obtained with the already in use method, proposed by the Italian Society of Compounding Pharmacists (SIFAP), was compared with two new methods, specifically the Tolotto Gear^®^ extraction method (TGE) and the Tolotto Gear^®^ extraction method preceded by a pre-extraction procedure (TGE-PE). HPLC analyses showed that the concentration of THC using cannabis flos with a high THC content (over 20% *w*/*w*) was always higher than 21 mg/mL for the Bedrocan variety and close to 20 mg/mL for the Pedanios variety when applying TGE, while with TGE-PE, the THC concentration was higher than 23 mg/mL for the Bedrocan variety. For the FM2 variety, the amounts of THC and CBD in the oil formulations obtained using TGE were higher than 7 mg/mL and 10 mg/mL, respectively, and for TGE-PE, the concentrations of THC and CBD were higher than 7 mg/mL and 12 mg/mL, respectively. GC-MS analyses were performed to define the terpene contents in the oil extracts. The samples of Bedrocan flos extracted with TGE-PE displayed a distinctive profile, highly rich in terpenes and devoid of oxidized volatile products. Thus, TGE and TGE-PE allowed performing a quantitative extraction of cannabinoids and increasing the total mono-di-tri terpenes and sesquiterpene concentrations. The methods were repeatable and applicable to any quantity of raw material, preserving the phytocomplex of the plant.

## 1. Introduction

**Figure 1:**
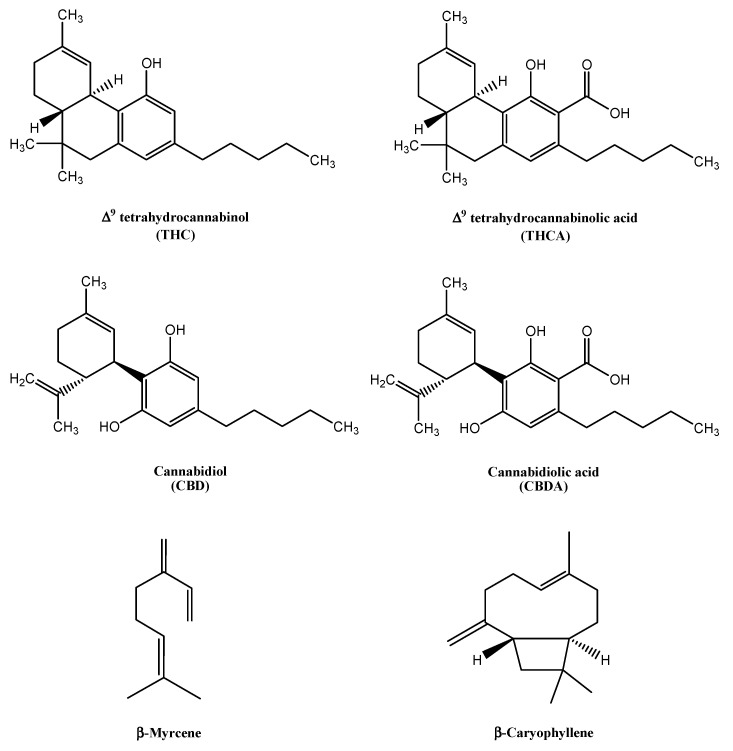
Molecular structures of the main cannabinoids Δ^9^-tetrahydrocannabinol (THC), Δ^9^-tetrahydrocannabinolic acid (THCA), cannabinol (CBD) and cannabinolic acid (CBDA), and the monoterpene β-myrcene and sesquiterpene β-caryophyllene.

*Cannabis sativa* L. is an annual cycle herbaceous flowering plant belonging to the Cannabinaceae family [1,2]. The plant is known to contain more than eight hundred compounds, among which the most important classes of active constituents are cannabinoids, terpenes, and flavonoids. Cannabinoids, a class of terpenophenolics [3], are abundantly produced in the glandular trichomes on female flowers [4]. Among them, the two most representative compounds are Δ^9^-tetrahydrocannabinolic acid (THCA) and cannabidiolic acid (CBDA) (Figure 1). These two acidic cannabinoids undergo a spontaneous decarboxylation under the action of light and heat, leading to formation of Δ^9^-tetrahydrocannabinol (THC) and cannabidiol (CBD) (Figure 1) [5].

THC, the main psychoactive constituent, is the most important active compound present in cannabis varieties used for either medical or recreational purposes [6]. CBD, present in fiber-type and medicinal *Cannabis sativa* varieties [6,7,8], also displays various pharmacological activities, such as antioxidant, anti-inflammatory, anti-microbial [9,10,11], and neuroprotective activity [12], related to the acting on several targets [9].

Terpenes are an additional well-represented class of active compounds produced in cannabis inflorescences [7,10]. Among terpenes, myrcene (Figure 1), limonene, trans-ocimene, and terpinolene are the most represented monoterpenes in the plant, while caryophyllene (Figure 1) and humulene are the most abundant sesquiterpenes [7,10]. Cannabis-based treatments represent a promising opportunity, as indications suggest a synergistic effect between cannabinoids and terpenes that is superior to synthetic drugs, known as the “entourage effect” [13,14,15,16,17].

Dried female flower tops of the plant are available as standardized medicinal grade material. The most common medical indications of cannabis include neuropathic pain [13]; chronic pain resistant to non-steroidal anti-inflammatory drugs, corticosteroids, or opioids; glaucoma resistant to conventional therapies [18]; undernutrition; chemotherapy-induced nausea and vomiting [19,20]; spasticity; and seizure in multiple sclerosis [20,21,22]. It is also a helpful therapeutic agent against cachexia and anorexia in patients with cancer or autoimmune disease syndrome and also in the reduction of body movements in Gilles de la Tourette syndrome [23].

Medical cannabis in Italy, as in the rest of the European Union, represents an irregular situation [15,24,25]. Dutch Bedrocan^®^ varieties (such as Bedrocan^®^, Bediol^®^, Bedica^®^, and Bedrolite^®^), and the two strains FM1 and FM2 produced by the Military Pharmaceutical Chemical Institute of Florence, Italy, can be prescribed together with other imported brands regulated by tenders of the Italian Ministry of Health [26]. Medical cannabis-based prescriptions are increasing in several countries, where therapeutic use is authorized; this is due to the positive role of cannabis in treating several pathological conditions, with few side effects [27,28]. Therefore, in many countries, pharmacists are legally allowed to formulate cannabis inflorescence doses for infusions, micronized capsules, vaping, and macerated oils [22,28,29,30].

In the case of pharmacy compounding, it is important to find extraction methods that allow the preservation of the complex cannabinoids/terpenes in products used for therapeutic purposes. Medical cannabis preparations can be used either orally or via inhalation [31]. As regards medical cannabis oils for oral therapeutic use, several extraction methods have been described in the literature [2,15,30,31,32,33,34]. Among them, in 2016 the Italian Society of Compounding Pharmacists (SIFAP) developed and proposed a method for preparing cannabis oil galenic extracts characterized by high yields [15]. The SIFAP method is a two-step procedure described for the preparation of a small oily extract batch, specifically 5 g of inflorescences extracted in 50 mL of pharmacopeia-grade olive oil, which does not allow controlling the entire decarboxylation process of cannabinoids.

In relation to the formulation of medical cannabis oils, no attention is generally paid to the terpene components of the plant. Indeed, the decarboxylation process, which is usually applied to the plant material to convert cannabinoids from the acidic into the neutral forms (Figure 1), can cause a complete loss of these volatile components. Since the method and conditions of cannabis oil preparation can have an impact on the composition of the formulation, the development and optimization of an efficient one-step procedure to be followed by pharmacists is important, to obtain a final formulation of high quality and to assure the reproducibility of its therapeutic outcomes. To our knowledge, none of the described procedures for cannabis oil preparation guarantee standardized oily extracts, in particular if applied to larger quantities of cannabis inflorescences, producing oleolites with a different content of active molecules. This limit is reflected by the difficulty in comparing clinical trials, in which extracts with different characteristics are often used.

In the light of all the above, in this study, with the aim of obtaining cannabis oily extracts rich in cannabinoids and terpenes, two extraction methods were evaluated. The extraction process was performed using an innovative technology, named Pharmagear^®^. This instrumentation integrated an automated process control system, able to control the temperature and the magnetic stirring during the extraction process, and a reactor (Tolotto) in which extraction and decarboxylation of the phytocomplexes occurred in a single-step.

## 2. Materials and Methods

### 2.1. Chemical and Solvent

In this study, several batches of *Cannabis sativa* L. varieties were used and purchased from different providers. Bedrocan (THC = 19–22%; CBD <1%) was purchased from the providers Farmalabor srl (Canosa di Puglia, BT, Italy), Fagron Italia srl (Quarto Inferiore, BO, Italy) and Galeno srl (Carmignano, PO, Italy). Cannabis Aurora Pedanios (THC 17–26%; CBD <1%) and FM2 (THC 5–8%; CBD 7.5–12%) were purchased from the Military Pharmaceutical Chemical Institute—Agenzia Industrie Difesa (Florence, FI, Italy). Caelo extra virgin olive oil (European Pharmacopoeia 9.3 (Eur. Ph. 9.3)) was purchased from Comifar Distribuzione SPA (Novate Milanese, MI, Italy). Cannabinoid standards (T-093-1 mL Δ^9^-Tetrahydrocannabinolic Acid; T-108-0.5 mL THC Cannabinoids Mixture-3; C-144-1 mL Cannabidiolic Acid (CBDA) used for chromatography were of analytical quality and were purchased from Merck (Milan, MI, Italy). Alpha-tocopherol (Eur. Ph.) was also purchased from Merck (Milan, MI, Italy).

### 2.2. Pharmagear^®^ Apparatus and Instruments

**Figure 2:**
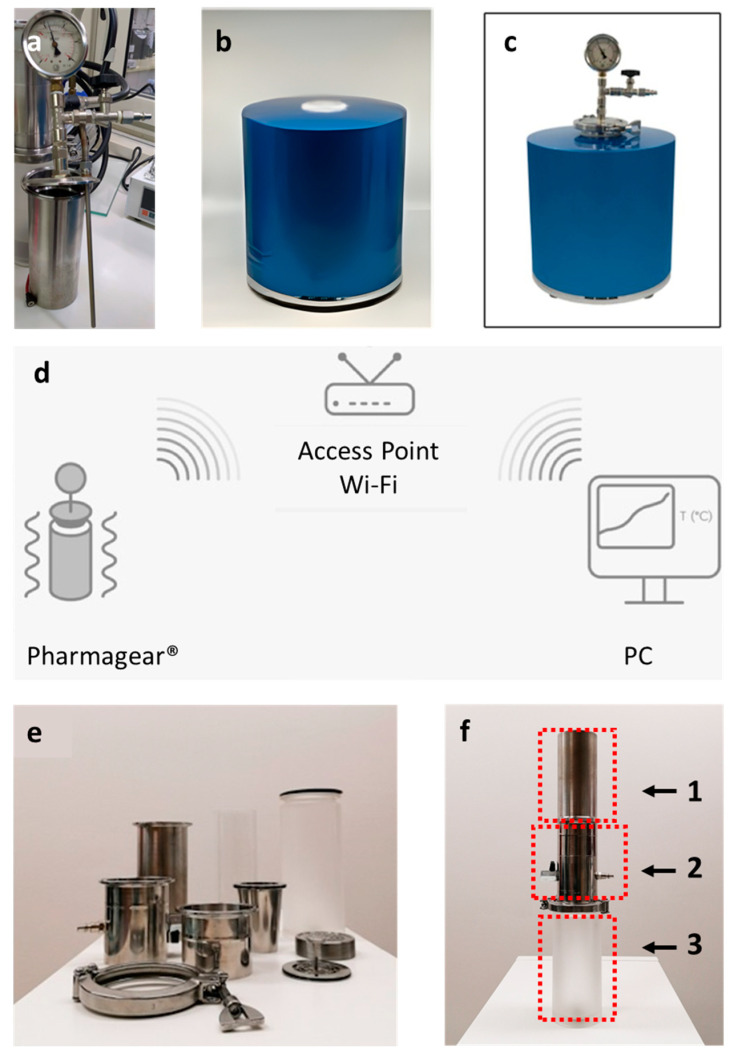
Tolotto, manometer, and vacuum valve (**a**); control system (**b**); Pharmagear^®^ (**c**); schematic representation of the automatic process of production of cannabis oils (**d**); filtration system tools (**e**); assembled filtration system constituted by the reactor (Tolotto) (1), connector for the vacuum pump (2), and glass cylindric container for the cannabis oil (3) (**f**).

For the preparation of cannabis oils, an innovative technology named Pharmagear^®^ (Energicamente srl, Favara, AG, Italy; Nebiolo Ht, Assoro, EN, Italy) was used (Figure 2). This instrument brings together a reduced pressure evaporator, an essential oil extractor constituted of a macerator coupled with an intelligent magnetic stirrer, and a reactor (Figure 2a–c). The extraction and decarboxylation processes are controlled by an automated electronic control system that sends in real time all the acquired data to software PharmaGear 1.0 (Energeticamente srl, Favara, AG, Italy) on a PC. This allows monitoring and controlling the entire decarboxylation process and measuring the CO_2_ produced during the heating phase with a specific CO_2_ probe (Figure 2d).

Times and temperatures are programmed using a software program before preparation. The system adapts its behavior according to pre-established temperatures, the preparation time, the phases of extraction and decarboxylation, the quantity of raw material to be heated, and environmental variables. The following equipment was also necessary for the preparation of the cannabis oils: Baoshishan FS-600 N Sonicator Ultrasonic Homogenizer 600 W Lab Sonicator Processor (Toption Instrument Co., Ltd., Xi’an, China); Turbo emulsifier Miccra Homogenizer (Riman SRL, Palermo, PA, Italy); vacuum pump filter system connected to the reactor.

### 2.3. Preparation of the Cannabis Extracts in Olive Oil

**Figure 3:**
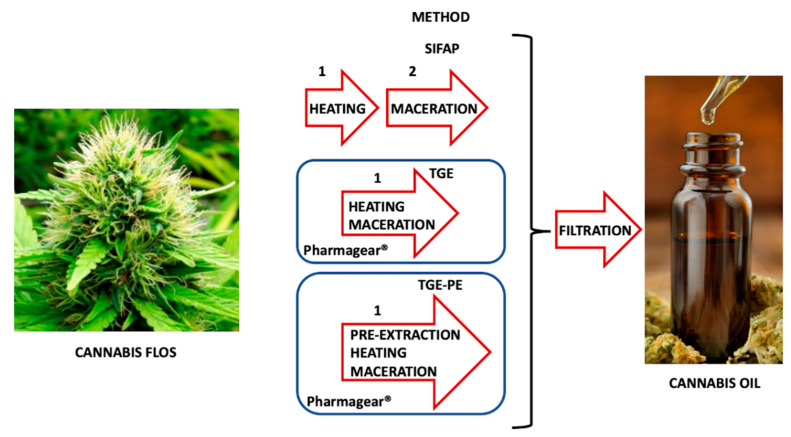
Schematic representation of the SIFAP procedure and TGE and TGE-PE methods for producing cannabis oil using Pharmagear^®^ technology.

Regardless of the quantities of cannabis to be extracted, for a minimum batch, 5 g of Cannabis flos were weighed and 50 mL of Ph. Eur. Olive Oil, previously cooled to a temperature between 2 and 8 °C, were measured. The reactor was wrapped with ice gel. A 10 mL aliquot of the oil was stored and added at the end of the entire process, for washing the reactor. The remaining 40 mL of Ph. Eur. Olive Oil was introduced to the reaction dish, together with the inflorescences, previously micronized, and dispersed in oil using a homogenizer. Any remaining cannabis inflorescences stuck in the rotor/stator head of the homogenizer were moved using a spatula and a second mixing cycle was performed for several minutes. Then, the mixture in the ice-gel-wrapped reactor was sonicated with a probe (Baoshishan FS-600 N Sonicator Ultrasonic Homogenizer 600 W Lab Sonicator Processor) at a delivered power of approximately 200 W for 5 min and frequency of 20 kHz. A magnetic anchor was inserted into the reaction dish, which in turn was closed with an airtight lid. The vacuum pump was connected to the reactor head and the oxygen was eliminated to reduce the oxidative stress of the oil during the subsequent heating phases. The reactor was assembled with a control system (Figure 2c) and using the Tolotto Gear^®^ extraction (TGE) method; two modes can be used: TGE alone or preceded by a pre-extraction procedure (TGE-PE). In particular, TGE consists in an extraction phase conducted at 110 °C for 120 min and a decarboxylation phase at 146 °C for 80 min, while TGE-PE requires a pre-extraction phase at room temperature (25–30 °C) for 12 h before starting the hot extraction process and subsequent decarboxylation, as described above for TGE (Figure 3). At the end of the process, the reactor was removed from the instrument and its temperature reduced to 40 °C with an ice gel wrap. The extracted cannabis oil was filtered using a food-grade nylon membrane filter system (pore size 37 μm) connected to a vacuum pump (VidaXL50 L/minpower 120 W, vacuum degree 50 Pa) (Figure 2f). The reactor and magnetic anchor were washed with the preserved oil fraction, which was added to the remaining oily preparation. The obtained cannabis oil was stored in an amber glass bottle, and alpha-tocopherol at 0.05% *v*/*v* was added to prevent oxidation. For batches with 10 and 15 g of plant material a 90- and 140-mL solvent volume of olive oil was used for the extraction process in the reactor, respectively. A 10 mL aliquot of oil was added at the end of the entire process to wash the reactor. In all the experiments, the drug:solvent volume ratio after dilution with 10 mL of washing solvent fraction was always maintained equal to 1:10.

### 2.4. Cannabis Extract in Olive Oil with the SIFAP Procedure (Maceration Process)

According to a method previously reported in literature [15], 5 g samples of *Cannabis sativa* L. were decarboxylated in an oven at 115 °C for 40 min. The plant material was then added to 50 mL of olive oil and further crumbled using a mixer. The extraction was performed at 100 °C over a period of 40 min, keeping an open beaker in a pre-heated and stirred silicone-oil bath. Then the mixture was immediately filtered, to obtain the final oil (Figure 3).

### 2.5. HPLC Instrument and Method

The HPLC instrument was an Agilent 1220 Infinity II LC gradient system VL equipped with a variable wavelength UV detector and openLab DCS software (Agilent Technologies, Waldbronn, Germany). An Agilent InfinityLab Poroshell 120 EC-C_18_ 3.0 × 50 mm, 2.7 µm column thermostated at 50 °C was used. Linear gradient elution (Table 1) with a flow rate of 1.0 mL min^−1^ was used. UV detection was carried out at 230 nm. An injection volume of 5 μL was used in all experiments. The analysis time was 11 min (with 1.5 min for re-equilibration).

#### Calibration Curve

Standard calibrators were produced from CBD, CBDA, THC, THCA, and cannabinol (CBN) certified reference standards, each at a concentration of 1.0 mg/mL in organic solvent. Equivalent volumes of each standard were mixed and diluted with methanol to a concentration of 250 µg/mL. Four dilutions were made, to generate a calibration curve at concentrations of 250, 100, 50, 10, and 1.0 µg/mL. Calibration curves were created as a response to the concentration and used for accuracy, precision, and linearity determinations, as described by Storm and colleagues. The analytical method used was that described in Agilent’s “dedicated cannabinoid potency test in cannabis or hemp” [35]. LOD (0.15 μg/mL) and LOQ (0.50 μg/mL) were determined using the signal-to-noise ratio (S/N) (Instrumental LOD: S/N = 3 and LOQ: S/N = 10). The standard solutions were stored away from light at a temperature of −20 °C until use.

### 2.6. Sample Preparation and Analysis of Cannabinoids and Terpenes

Qualitative and quantitative analysis of the profile of cannabinoids in the cannabis oil formulations was carried out using the HPLC method described in Section 2.5. HPLC instrument and method. First, 50 µL or 500 µL aliquots of each homogenized concentrated cannabis oil were pipetted into a calibrated volumetric flask and diluted to a final volume of 5 mL with high purity HPLC grade ethanol. Then, 2 mL of each solution was filtered using a glass syringe fitted with a 0.45 μm regenerated cellulose syringe filter. An additional 10-fold dilution of the filtered solution was performed by transferring a 100 µL aliquot into an amber glass 2 mL auto-sampler vial and adding 900 µL high purity HPLC grade methanol. Then, the prepared samples were analyzed by HPLC.

The determination of terpene profile was carried out according to the method developed by Aiello and coworkers for the evaluation of volatile organic compounds [36].

### 2.7. Statistical Analysis

Concentrations of cannabinoids and terpenes in the analyzed samples were expressed both as mean values and related standard deviation (S.D.). Statistical analyses were performed using GraphPad Prism 5.0 (GraphPad Software, Inc., La Jolla, CA, USA). In order to reveal potential discriminating features between the groups, a one-way analysis of variance (ANOVA) test was performed. Bonferroni’s multiple comparison test (MCT) was applied as a post hoc test. The groups were designed considering the cannabis varieties (Bedrocan, Pedanios, and FM2) and extraction protocol (TGE, TGE-PE, and SIFAP). Significant variables were expressed by a *p*-value with a threshold < 0.05.

## 3. Results and Discussions

The preparation of pharmaceutical grade olive oil formulations of *Cannabis sativa* L. for medical use usually requires a decarboxylation step followed by an extraction procedure. The latter, called the SIFAP method, is one of the most widely used. In this study, the application of a new one-step technology aimed at protecting the phytocomplex from degradation during the extraction procedure was studied. By means of a new technological platform, named Pharmagear^®^, two methods, named TGE and TGE-PE, for the preparation of pharmaceutical grade olive oil formulations were compared, in terms of yields of extracted cannabinoids and terpenes.

**Figure 4:**
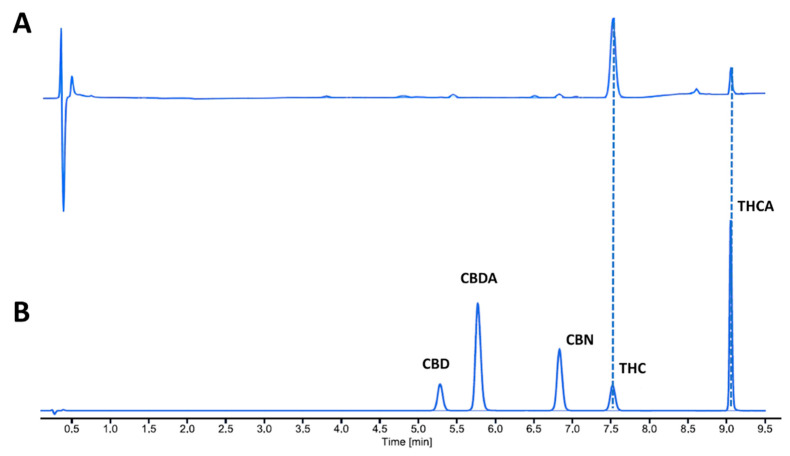
Representative HPLC chromatogram of Bedrocan oil formulation obtained using TGE method (**A**), and calibration chromatogram of standards for analytes used in the study (**B**).

Cannabinoid quantification was obtained using HPLC, and a representative chromatogram is reported in Figure 4.

Table 2 shows the amounts of THC, THCA, CBN, CBD, and CBDA obtained using the TGE and TGE-PE methods. At the beginning of 2022, the Pedanios variety was not supplied by any provider to complete the study using TGE-PE; therefore, it was only possible to carry out experiments using TGE. The concentrations (mg/mL) of the active ingredients are expressed as mean values with their standard deviation (S.D.). Cannabinoid concentrations were found to be in line with data available in the literature for each vegetal material preparation [2,28,31].

Since the extractive yield and decarboxylated products was always close to 100% of the achievable result (considering that Bedrocan and Pedanios have a declared THC content in the plant (sum of THC and THCA) close to or higher than 20% *w*/*w* and FM2 has a declared content of THC in the plant in a range of 5–8% *w*/*w* and of CBD 7.5–12% *w*/*w*), overall the TGE and TGE-PE methods appeared almost equivalent in terms of the cannabinoids extracted (Table 2). These results were compared to those obtained using the SIFAP method, one of the most widespread high-capacity extraction methods. The comparison was performed on the basis of data reported in the literature and a new experimental dataset.

Over the period from 2017 to 2019, hundreds of samples were prepared by pharmacists and analyzed by the University of Milan [34]. Despite a THC content close to or greater than 20% *w*/*w*, an average extraction of 12.236 ± 3.31 mg/mL was obtained using the Cannabis flos varieties Bedrocan and Pedanios (*n* = 800), while residual THCA was less than 1.8 mg/mL. In the case of FM2 (*n* = 350), the THC mean value was 5.06 ± 1.01 mg/mL and CBD was 7.268 ± 1.84 mg/mL (Table 3).

The extraction capacities of the two proposed methods (Table 2) using the innovative platform were almost doubled compared to those described in the literature [34]. The concentration of THC using cannabis flos with high THC content (over 20% *w*/*w*) was always higher than 21 mg/mL for the Bedrocan variety and close to 20 mg/mL for the Pedanios variety when applying the TGE method, while for with TGE-PE method used with the Bedrocan variety, the THC concentration was higher than 23 mg/mL. A significant difference (*p* value < 0.01) was found between the THC quantities in oil formulations from the Bedrocan variety obtained when applying the TGE and TGE-PE methods. Moreover, for FM2, the amounts of THC and CBD in the oil formulations obtained using the TGE method were higher than 7 mg/mL and 8 mg/mL, respectively (Table 2), and for the TGE-PE method, the concentrations of THC and CBD were higher than 7 mg/mL and 12 mg/mL, respectively (Table 2). The THC quantities extracted from the FM2 variety with both TGE and TGE-PE methods were higher and statistically significant (*p* values < 0.01) than the mean values reported in literature [34] (Table 3), while the CBD quantities extracted from the FM2 variety with only TGE-PE method were statistically significant (*p* values < 0.01) compared to the SIFAP results. No significant differences between the THC contents in oil formulations produced using different amounts of cannabis flos and olive oil (5:50, 10:100 and 15:150, Table 2) were observed. The results of the tests carried out with three different quantities of plant material support that the methods are also suitable for multiple batches. Some differences in terms of the amounts of THCA and CBDA determined in the HPLC analysis using different amounts of cannabis flos and oil could be attributed to the residual contents of these cannabinoids in the various batches of plant materials purchased from different suppliers.

To confirm the advantages offered by the new platform Pharmagear^®^, the TGE method was compared with new experimental SIFAP results (maceration process). Table 4 shows the content in mg/mL of cannabinoids in the oil formulations after extraction of 5 g of the cannabis flos variety Bedrocan.

As one can see from Table 4, the obtained experimental results were in agreement with the data reported in the literature [34]. The THC content in the oil formulation produced using the TGE method was significantly different (*p* < 0.01) from the content in the formulation obtained using the maceration process.

The characteristic scent of cannabis is the result of the presence of about 140 different terpenes and terpenoids. Terpenes are a mixture of different compounds consisting of multiples of the isoprene unit (C_5_H_8_). β-myrcene, limonene, trans-ocimene, and α-terpinolene are the most abundant monoterpenes in cannabis inflorescences, while β-caryophyllene and α-humulene are the most represented sesquiterpenes. These compounds have antioxidant, anti-inflammatory, anxiolytic, and anti-bacterial properties. During the preparation process of most oleolites produced with various methods, the heating that is applied to the plant material to convert the acidic cannabinoids into neutral compounds greatly reduces the lighter terpene percentage (monoterpenes). The use of high temperatures increases the concentration of sesquiterpenes, to the detriment of monoterpenes. To overcome this problem, the TGE method was designed to perform an extraction in a hermetic environment, so as to allow the condensation of the terpenes after cooling and under vacuum, in order to eliminate the oxidative stress of the oil during heating. In this study, a preliminary investigation into the ability of the TGE and TGE-PE methods to extract and preserve terpenes using the innovative technology Pharmagear^®^ was conducted. TGE-PE differs from the former because it uses a pre-extraction of 12 h at a temperature of 30 °C.

The concentrations in mg/Kg of the terpenes extracted from Bedrocan flos are presented in Table 5. The concentrations of terpenes were found to be in line with the data available in the literature for oil preparations [25,31]. The quantity of preserved terpenes can be used as an indication of preservation of the phytocomplexes.

Mono-di-tri terpenes were highly represented in the analyzed sample (Table 5). Overall, the two methods showed a different performance, in terms of the final quantitative yield of the extracted terpenes. On the other hand, the sample of Bedrocan flos extracted with the TGE-PE method displayed a distinctive profile, highly rich in terpenes and devoid of oxidized volatile products. The quantified molecules belonging to this chemical class were α-pinene, limonene, and β-caryophyllene, showing a significant increasing trend when performing pre-extraction procedures compared to the TGE method. TGE-PE showed a greater extraction capacity in terms of the terpenes obtained. About 6% more active ingredients were extracted than the amount extracted using the TGE method (Table 5), even if for the less representative terpenes, in terms of concentration, this difference was amplified. Moreover, some terpenes could only be extracted using the TGE-PE method (Table 5). Cis-p-menth-2,8-dienol, α-limonene diepoxide, α-guaiene, and 5-caranol (Table 5) were only present in the oil formulation obtained using the TGE-PE method and absent in the extraction with the TGE method.

Using the new platform, the two proposed methods allow preserving the terpene fractions, with no increase in sesquiterpenes to the detriment of monoterpenes.

During the decarboxylation process, it was possible to observe an increase in the internal pressure of the bath, with consequent compensation for the negative pressure, due to the formation of CO_2_. This phenomenon is an indication of decarboxylation. The total cooling of the Tolotto before its opening is decisive and is optimized to allow the condensation and recovery of the terpenes, which otherwise would be lost. Moreover, the very low concentration of CBN indicated a low degradation of THC, and together with the high THC values, this confirmed the validity of the methods. The cooling of the Tolotto allowed the condensation of the terpenes and its tightness determined the redissolution of the vapor phase in the solvent according to Henry’s law (a gas that exerts pressure on the surface of a liquid enters the solution until it has reached the same pressure it exerts on it). By increasing the pressure above the liquid proportionally, this increased the quantity of gas that passed into solution. The division of the methods into two parts, extraction and decarboxylation in the Tolotto, is purely ideal, in the sense that part of the decarboxylation occurred during extraction and vice versa.

The new technology described in this study and the two extraction methods used are very promising for both procedures for preparation of an extemporaneous oil formulation from *Cannabis sativa* L. for medical use and the standardization of the chemical composition of the bioactive molecules in extracts, and especially for the presence of terpenes (as one can see from the preliminary results in Table 5). Among these, limonene, pinene, myrcene, and β-caryophyllene, compounds known to be individually responsible for the anti-inflammatory, analgesic, antinociceptive, anxiolytic, and synergistic effects with phytocannabinoids [17,37,38], were present in a high concentrations in the oleolites produced using the TGE and TGE-PE methods.

In this context, a differential cannabinoid receptor activity and the inhibition of glycoprotein-P [39,40] by terpenes have been studied and established. Moreover, many studies have demonstrated terpene enhancement of the absorption of several active pharmaceutical ingredients, such as analgesics and anti-inflammatories [41,42,43,44,45,46]. In addition, a cannabis formulation containing essential terpenes had a multi-target synergy [47]. In fact, each component of the phytocomplex could exert low therapeutic potency, but their concurrent pharmacological action was shown to be highly effective and characterized by a low toxicity [48]. Thus, it is important to formulate cannabis oil preparations characterized by a high cannabinoid and terpene content.

## 4. Conclusions

To standardize the extraction conditions for medical cannabis oily preparations, SIFAP offers a suitable procedure to pharmacists. Several methods have also been proposed and adopted, but all of them are based on two separate steps: extraction and decarboxylation processes. The TGE and TGE-PE methods, based on an innovative automatic platform, allowed the extraction of the phytocomplex in a single step, controlling the entire decarboxylation process of cannabinoids. HPLC analyses performed on the oil formulations showed that the extraction capacities of the two proposed methods using the innovative platform were almost double compared to those obtained using the SIFAP procedure. The content of THC in the oil formulations was higher than 21 mg/mL for the Bedrocan variety and close to 20 mg/mL for the Pedanios variety when applying TGE, while with TGE-PE, the THC concentration was higher than 23 mg/mL for the Bedrocan variety. For the FM2 variety, the amounts of THC in the oil formulations obtained using TGE and TGE-PE were higher than 7 mg/mL. GC-MS analyses of the oil formulations obtained from Bedrocan flos extracted with TGE-PE displayed a distinctive profile highly rich in terpenes and devoid of oxidized volatile products. Thus, the application of TGE and TGE-PE extraction methods permitted producing standardized oily formulations with an increased composition of cannabinoids and total mono-di-tri terpenes and sesquiterpene concentrations.

The results obtained are relevant and allow exploring a new way of thinking about preparations in the galenic laboratory. Modern and accurate preparation process control systems establish the entrance of the Technology of Industry 4.0 or “Smart Factory” into the galenic laboratory and lend themselves to further application developments. The automation and accuracy of the system does not require the continuous control of an operator during the preparation phases and allow for accuracy, repeatability, and the avoidance of human error. The developed equipment proved useful, not only for the preparation of oil, but also for decarboxylated capsules based on medical cannabis. Based on the initial evaluation of the results obtained through this new technology, further studies will be conducted, in consideration of the fact that the literature on the terpene profile present in extracts of oily medical cannabis is very scarce.

## 5. Patent

An Italian patent was granted by the Italian Office for Patent and Brands for the procedure for Cannabis oil production (patent number 102019000014901, 3 August 2021).

## Figures and Tables

**Table 1:** HPLC mobile phase gradient.

Times (min)	0.1% (*v*/*v*) Formic Acid Aqueous Phase	0.05% (*v*/*v*) Formic Acid in Methanol
0	40	60
1.0	40	60
7.0	23	77
8.2	5	95
9.5	40	60
11.0	40	60

**Table 2:** Concentrations of the active ingredients (mg/mL) after extraction of Bedrocan, FM2, and Pedanios medical plant material using the TGE and TGE-PE methods (*n* = 3; mean value ± S.D.).

Compound	Plant Material (g) in Solvent Volume (mL) *	Bedrocan (mg/mL) (Mean ± S.D.)		FM2 (mg/mL) (Mean ± S.D.)		Pedanios (mg/mL) (Mean ± S.D.)
		TGE	TGE-PE	TGE	TGE-PE	TGE
THC	5:50	21.084 ± 0.066	23.940 ± 2.310	7.811 ± 1.640	6.686 ± 0.810	20.784 ± 0.701
	10:100	21.704 ± 2.170	23.215 ± 0.380	7.107 ± 0.535	6.894 ± 0.960	19.584 ± 1.420
	15:150	22.259 ± 1.420	23.986 ± 0.900	7.223 ± 0.880	7.758 ± 0.590	19.590 ± 0.481
THCA	5:50	1.000 ± 0.416	1.126 ± 0.470	0.172 ± 0.170	N.Q.	0.548 ± 0.434
	10:100	1.526 ± 1.130	1.555 ± 0.120	0.218 ± 0.380	N.Q.	2.521 ± 0.130
	15:150	1.140 ± 0.323	0.254 ± 0.260	N.Q.	N.Q.	2.012 ± 1.460
CBD	5:50	N.Q.	N.Q.	8.093 ± 1.480	12.126 ± 0.430	N.Q.
	10:100	N.Q.	N.Q.	10.712 ± 0.740	11.847 ± 0.830	N.Q.
	15:150	N.Q.	N.Q.	12.424 ± 0.280	13.217 ± 0.830	N.Q.
CBDA	5:50	N.Q.	N.Q.	1.386 ± 1.220	N.Q.	N.Q.
	10:100	N.Q.	N.Q.	0.768 ± 1.350	0.685 ± 1.190	N.Q.
	15:150	N.Q.	N.Q.	0.396 ± 0.690	N.Q.	N.Q.
CBN	5:50	0.150 ± 0.136	0.214 ± 0.370	N.Q.	0.264 ± 0.460	0.390 ± 0.265
	10:100	0.169 ± 0.140	0.299 ± 0.520	N.Q.	0.485 ± 0.420	0.301 ± 0.065
	15:150	0.260 ± 0.133	N.Q.	N.Q.	N.Q.	0.298 ± 0.084

N.Q.: not quantifiable; * Solvent volumes include the 10 mL olive oil used for washing the reactor.

**Table 3:** Concentration of the active ingredient cannabinoids (mg/mL) expressed as mean value determined over the period from 2017 to 2019 by analyzing 800 samples for Cannabis flos varieties (Bedrocan and Pedanios) with a content of THC (sum of THC and THCA) close to or higher than 20% *w*/*w,* and 350 samples for a Cannabis flos variety (FM2) with a content of THC (sum of THC and THCA) between 5 and 8% *w*/*w* and CBD between 7.5 and 12% *w*/*w*.

Compound	Bedrocan/Pedanios (mg/mL) (Mean ± S.D.)	FM2 (mg/mL) (Mean ± S.D.)
THC	12.236 ± 3.31	5.06 ± 1.010
THCA	<1.840	N.Q.
CBD	N.Q.	7.268 ± 1.840

N.Q.: not quantifiable.

**Table 4:** Concentrations of active ingredients (mg/mL) after extraction of Bedrocan medical plant material (5 g) using the TGE and SIFAP methods. The results were determined through analysis of the chromatograms of the oil formulations with final volumes of 50 mL (*n* = 3; mean value ± standard deviation, S.D.).

Compound	TGE (mg/mL) (Mean ± S.D.)	SIFAP (mg/mL) (Mean ± S.D.)
THC	23.037 ± 1.956	16.719 ± 1.330
THCA	1.263 ± 0.871	1.711 ± 0.504
CBD	N.Q.	N.Q.
CBDA	N.Q.	N.Q.
CBN	0.376 ± 0.342	0.320 ± 0.231

N.Q.: not quantifiable.

**Table 5:** Terpenes in oil formulations of Bedrocan flos. Concentrations of active ingredients (mg/Kg) after extraction of 5 g of medical plant material using the TGE and TGE-PE methods. The quantities (mean values using duplicate measurements) were determined through GC-MS analysis of an oil formulation with a final volume of 50 mL.

Terpenes	TGE (mg/Kg)	TGE-PE (mg/Kg)
α-copaene	9.14	18.82
nerolidol	17.90	25.64
cis-geraniol	20.96	37.83
ylangene	25.63	45.67
α-bergamotene	29.26	42.60
τ-gurjunene	30.95	48.00
borneol	38.54	48.16
iso-caryophyllene	40.09	59.06
α-gurjunene	41.08	48.0
α-selinene	47.23	72.70
eremophilene	49.04	104.72
trans-3-caren-2-ol	49.53	83.68
α-farnesene	55.27	47.43
β-farnesene	66.83	728.77
β-selinene	69.90	139.93
τ-selinene	71.88	72.70
fenchol	85.03	108.87
β-phellandrene	85.86	112.17
2-pinanol	105.75	193.08
p-mentha-1,3,8-triene	106.46	199.73
β-trans-ocimene	112.94	111.49
aromadendrene	118.34	249.64
α-thujene	129.13	129.14
δ-guaiene	245.52	374.41
τ-terpinene	271.95	299.21
α-pinene	307.47	371.09
3-carene	308.99	313.79
selina-3,7-diene	350.59	532.26
α-humulene	354.81	677.37
β-pinene	373.57	439.82
guaia-3,7-diene	377.59	560.34
cis-carveol	393.69	557.70
α-terpinene	402.40	371.84
myrtenol	418.47	460.76
α-phellandrene	443.89	482.53
p-cymene	501.84	493.83
linalol	595.96	689.23
p-cymen-8-ol	738.33	1421.52
α-terpineol	1109.98	1507.06
caryophillene	1224.36	1762.26
limonene	1358.72	1431.70
β-cis-ocimene	3650.65	3821.75
β-myrcene	5144.46	5457.31
α-terpinolene	8544.46	9038.39
cis-*p*-menth-2,8-dienol	N.Q.	71.71
α-limonene dieposside	N.Q.	82.25
α-guaiene	N.Q.	109.64
5-caranol	N.Q.	117.39
**Total Concentration**	**28,524.44**	**33,945.96**

N.Q.: not quantifiable.

## Data Availability

Not applicable.
